# Editorial: Understanding the impact of microbes on tumor progression and prevention: unveiling new avenues for cancer therapy

**DOI:** 10.3389/fimmu.2025.1705365

**Published:** 2025-10-13

**Authors:** Vikas Kumar Somani, Somya Aggarwal, Rajni Garg, Yoshiko Takeuchi, Samer Singh

**Affiliations:** ^1^ Washington University School of Medicine, St. Louis, MO, United States; ^2^ Department of Human Genetics and Molecular Medicine, Amity School of Health Sciences, Amity University, Mohali, Punjab, India; ^3^ Institute of Medical Sciences, Banaras Hindu University, Varanasi, India

**Keywords:** Microbiome, microbes, cancer, ovarian cancer, colon cancer, cutaneous cancer, T-cell lymphoma

The intricate interplay between the human microbiome and host physiology has emerged as a pivotal area of biomedical research. Microbial communities, once regarded as passive colonizers, are now recognized as active modulators of diseases, such as cancer. This Research Topic, *“Understanding the Impact of Microbes on Tumor Progression and Prevention: Unveiling New Avenues for Cancer Therapy,”* compiles studies illuminating the diverse roles played by microbes in shaping tumor biology. Together, these contributions highlight the dualistic nature of microbes as both facilitators and inhibitors of tumorigenesis and underscore their potential as therapeutic targets, biomarkers, and modulators of treatment responses.

The Research Topic begins with a comprehensive review by Lin and Zhang. This work systematically evaluated bacterial contributions to the metastatic cascade. The authors illustrated how bacteria promote tumor spread by inducing chronic inflammation, modulating the extracellular matrix, and enabling tumor cells to evade immune surveillance. At the same time, probiotics and engineered bacterial strains showed the ability to suppress metastasis, evidencing the bidirectional nature of microbial influence on cancer progression. This duality exemplifies the broader theme of the Research Topic, i.e., microbes as both potential drivers and therapeutic allies in oncology.

Focusing on ovarian cancer, Zhang et al. explored how microbial alterations modulate local inflammation within the tumor microenvironment^2^. Their analysis highlighted the importance of microbial dysbiosis in sustaining pro-inflammatory signals that support tumor growth. By linking microbial shifts with immune modulation, the article emphasizes a translational opportunity: leveraging microbiome-targeted interventions for both therapeutic development and early screening. This mechanistic perspective positions microbial–immune interactions as a focal point for future ovarian cancer strategies.

The relationship between microbes and cancer therapy was further illustrated by Chrisman et al., in *“Ionizing radiation improves skin bacterial dysbiosis in cutaneous T-cell lymphoma”* Their longitudinal study demonstrated that total skin electron beam therapy (TSEBT), a standard treatment for CTCL, exerts effects beyond cytoreduction. The radiotherapy restored the balance of the microbial community, reducing the dominance of *Staphylococcus aureus* and facilitating the recolonization of beneficial commensals. These findings propose an additional therapeutic dimension: radiotherapy may act in part by correcting microbial dysbiosis and improving skin barrier function. By situating microbiome restoration within the context of cancer treatment, this study broadens our understanding of radiotherapy’s multifaceted mechanism of action^3^.

In line with the theme of microbial modulation of therapy, De Re et al. demonstrated in their study that virulent *H. pylori* strains can disrupt host DNA repair pathways while promoting immune evasion via PD-L2 upregulation. This microbial interference could undermine the efficacy of HER2-targeted treatments, complicating therapeutic predictability. Importantly, their findings raise the possibility that unaddressed microbial infections may influence targeted therapy outcomes, emphasizing the need to integrate microbial status into clinical decision-making for gastric cancer.

Further insight into microbe-driven cancer progression was provided by Li et al., who investigated the role of *Fusobacterium nucleatum* in colorectal cancer^5^. Their study identified Serpine2 as a key factor, which is upregulated by bacterial influence, and promotes fibroblast–epithelial communication and tumor progression. These observations highlight microbial shaping of stromal dynamics, suggesting that disrupting this microbe-induced crosstalk could provide a novel therapeutic strategy in colorectal cancer.

Broadening the perspective beyond individual cancer types, Liu et al. offered a pan-cancer analysis in their study^6^. By evaluating 783 tumor samples from seven cancer types, their study revealed widespread intratumoral microbial presence, with recurring genera such as *Pseudomonas, Streptococcus*, and *Prevotella.* Moreover, microbial diversity analyses demonstrated significant differences between cancerous and adjacent healthy tissue, particularly in gastric and liver cancers. These findings identify intratumoral microbial signatures as potential pan-cancer biomarkers with diagnostic and prognostic value, suggesting new paths toward microbial signature-based stratification in cancer management.

Taken together, these contributions present a coherent framework positioning the microbiome as a critical determinant of tumor biology. At the mechanistic level, microbes may influence tumorigenesis by modulating inflammation, evading immune responses, reprogramming stromal cells, and interfering with cell signaling and repair pathways. On a translational level, microbial signatures are emerging as potential biomarkers for diagnosis, prognosis, and therapeutic response. Importantly, microbial modulation—via radiation, probiotics, engineered strains, or targeted therapies—offers novel possibilities for augmenting standard cancer treatments.

Several themes have emerged from this Research Topic. First, microbial influences are context-dependent, exerting either pro- or anti-tumorigenic effects depending on host state, cancer type, and microbial composition. This underscores the necessity of finely tuned therapeutic strategies that proactively consider microbial ecology. Second, cancer therapies themselves—whether radiation or immune-targeted agents—may shift microbial communities, creating feedback loops that influence clinical outcomes. Finally, at the systems level, conserved microbial signatures across multiple tumor types suggest the possibility of universal microbial biomarkers or targets, opening up the opportunities for broad-spectrum approaches in precision oncology.

The implications of these findings are profound. There is a growing recognition that cancer is not just a disease of malignant cells but rather a product of ecological networks that include microbial partners. Therapeutic modalities that acknowledge this broader ecosystem—through microbiome correction, exploitation, or monitoring—could transform cancer care. Importantly, future research must integrate longitudinal, mechanistic, and patient-centered approaches to clarify causal relationships and define actionable strategies.

In summary, this Research Topic of seven studies illustrates the breadth and depth of microbial involvement in cancer (**Table 1**). From ovarian to colorectal and cutaneous malignancies, from localized therapeutic effects to pan-cancer microbial patterns, the evidence consistently highlights microbes as integral elements of the tumor microenvironment. By unraveling these complex host–microbe interactions, we are moving toward a paradigm in which the microbiome is no longer peripheral but central to cancer biology and therapy. As the field advances, microbial signatures and interventions could form the foundation for next-generation diagnostic and therapeutic platforms, ultimately bringing us closer to personalized and effective strategies for cancer prevention and treatment.

**Table 1:** Overview of contributions to the Research Topic.

Therapeutic Target/Strategy	Mechanism/rationale	Cancer type(s)	Contributing article
Microbiome-Targeted Therapies	Modulating the tumor’s inflammatory microenvironment by altering its microbial composition.	Ovarian Cancer	Zhang et al.
Gut and Intratumoral Microbiota as Biomarkers	Using microbial signatures to predict response to triple therapy (lenvatinib + PD-1 inhibitors + local therapy).	Hepatocellular Carcinoma (HCC)	Lin et al.
Restoration of the Skin Microbiome	Using therapies such as ionizing radiation to reverse dysbiosis, reduce pathogenic bacteria (*S. aureus*), and increase beneficial commensals.	Cutaneous T-cell Lymphoma (CTCL)	Chrisman et al.
PD-L2 and DNA Repair Pathways	Targeting immune evasion pathways (PD-L2) and DNA repair machinery (MSH6), which are modulated by *H. pylori* infection.	HER2-Positive Gastric Cancer	De Re et al.
Serpine2 Molecule	Inhibiting Serpine2, a key molecule that is upregulated by *F. nucleatum* and enhances fibroblast-epithelial cell communication to promote tumor progression.	Colon Cancer	Li et al.
Probiotics and Engineered Bacteria	Utilizing beneficial microbes to inhibit metastasis by modulating the immune response and remodeling the tumor microenvironment.	General (Review)	Lin and Zhang
Pan-Cancer Microbial Diversity as Biomarkers	Using intratumoral microbial diversity indices (Shannon, Simpson) and common bacterial genera (Pseudomonas, Streptococcus, and Prevotella) for the diagnosis and prognosis of multiple cancer types.	Multiple Cancers (Breast, Esophageal, Gastric, Liver, Lung, Pancreatic, OSCC)	Liu et al.

